# UFMylation orchestrates kidney development through YAP stabilization

**DOI:** 10.1016/j.jbc.2026.113285

**Published:** 2026-06-22

**Authors:** Yi Chen, Longyi Wan, Wei Chen, You Zhou, Fuzhao Zhang, Junyan Zhang, Chenlu Zhang, Qian Liang, Yu Jiang, Ying Wang, Yajun Duan, Yu-Sheng Cong, Jiang Liu

**Affiliations:** 1 Zhejiang Key Laboratory of Medical Epigenetics, Department of Cell Biology and Genetics, School of Basic Medical Sciences, Hangzhou Normal University, Hangzhou, Zhejiang, China; 2 Department of Cardiology, The First Affiliated Hospital of USTC, Division of Life Sciences and Medicine, University of Science and Technology of China, Hefei, Anhui, China

**Keywords:** kidney dysplasia, UFL1, ufmylation, UFSP1, UFSP2, YAP

## Abstract

The ubiquitin-like modifier UFM1 regulates diverse cellular processes through UFMylation. We previously demonstrated that UFMylation ligase UFL1 deficiency induces endoplasmic reticulum stress and culminates in kidney atrophy in adult mice, yet the critical UFMylation substrates and its function in organogenesis remain largely unexplored. Here, we report UFMylation is required for mammalian kidney development through Yes-associated protein (YAP) stabilization. Genetic ablation of *Ufl1* in the murine kidney results in severe dysplasia, phenocopying the loss of YAP activity. We identified YAP as a substrate for UFMylation at lysine 76 (K76), which shields YAP from ubiquitin-proteasome degradation, thereby promoting its nuclear accumulation and transcriptional activation. Additionally, we discovered UFSP1, rather than UFSP2, acted as the primary protease for YAP de-UFMylation, whose depletion promoted YAP stabilization and activation. These findings establish UFMylation as a crucial post-translational mechanism licensing YAP-dependent transcription during development, reveal unexpected complexity in the regulation of YAP UFMylation, and imply a previously unrecognized etiological pathway for kidney dysplasia.

UFMylation is a newly identified ubiquitin-like modification that orchestrates a broad spectrum of cellular functions through covalently conjugate UFM1 to substrate proteins (1, 2, 3). Similar to ubiquitination, UFMylation proceeds an enzymatic cascade involving the E1 activating enzyme UBA5, the E2 conjugating enzyme UFC1, and the E3 ligase UFL1. DDRGK1 (also known as UFBP1) interacts with UFL1 to form an obligate heterodimer required for UFL1 ligase activity (4, 5). UFMylation is also dynamically reversible, with UFSP1 and UFSP2 serving as proteases responsible for both UFM1 maturation and de-UFMylation of substrates (6, 7, 8). To date, a growing number of UFMylation substrates are identified and well characterized, such as activating signal cointegrator 1 (ASC1) (4), ribosomal protein L26 (RPL26) (9, 10), meiotic recombination 11 (MRE11) (11), histone H4 (12), p53 (13), retinoic acid-inducible gene 1 (RIG-1) (14), NADH-cytochrome b5 reductase 3 (CYB5R3) (15), programmed death ligand-1 (PD-L1) (16), PD-1 (17), PARP1 (18), and actin-related proteins 4 (ARPC4) (19), which led to diverse cellular processes and linked to a variety of diseases, ranging from metabolic diseases to cancer, hip dysplasia, heart failure, and neurodevelopmental disorders (20, 21, 22). Although these findings underscore the broad relevance of UFMylation in human disease, its role in tissue homeostasis remains poorly understood. We previously demonstrated that ablation of *Ufl1* in the kidney leads to renal atrophy *via* disruption of endoplasmic reticulum homeostasis (23), yet the critical downstream substrates mediating UFMylation-dependent tissue maintenance needs further investigation.

Yes-associated protein (YAP) is a pivotal transcriptional coactivator that governs organ size, tissue homeostasis, and embryonic development (24, 25, 26). During renal morphogenesis, YAP is activated from the earliest stages and persists throughout organogenesis (27, 28, 29). Consistent with its essential role, conditional knockout of *Yap* in the kidney results in profound hypoplasia and neonatal lethality in mice (30). As a key component of the Hippo signaling pathway, YAP is inhibited by the mammalian STE20-like kinase half (MST1/2)-large tumor suppressor kinase half (LATS1/2) kinase cascades of the Hippo pathway through phosphorylation (31, 32). Phosphorylation at YAP serine 127 (S127) results in its cytoplasmic retention by binding with 14-3-3 protein (33), while phosphorylation at S397 primes it for further phosphorylation by casein kinase 1δ/ϵ (CK1δ/ϵ), creating a phosphodegron that recruits the SCF^β-TRCP^ E3 ubiquitin ligase and targets YAP for proteasomal degradation (34, 35). Beyond development, YAP regulation is also implicated in stem cell maintenance and tumorigenesis, underscoring its broad physiological and pathological significance (36, 37, 38). The precise control of YAP stability and activity through diverse post-translational modifications thus represents a fundamental regulatory node, making it an attractive and challenging therapeutic target.

In this study, we discovered that renal tubule-specific ablation of *Ufl1* led to severe kidney dysplasia, extending beyond the kidney atrophy. *Ufl1* deficiency enhanced the interaction between LATS1/2 and YAP, and led to YAP hyperphosphorylation, which resulted in functional inactivation of YAP and abrogation of its pro-developmental gene programs. Mechanistically, we identified YAP as a *bona fide* substrate for UFMylation at lysine 76 (K76), which protected YAP from ubiquitin-proteasome degradation, thereby enabling its nuclear accumulation and transcriptional activation. A striking divergence was observed between the two de-UFMylating proteases: UFSP2 deficiency enhanced YAP phosphorylation and degradation, whereas UFSP1 deficiency promoted YAP stabilization and activation, indicating that UFSP1 serves as the dominant protease for YAP deconjugation. Thus, we unveil a UFMylation-dependent regulatory axis, orchestrated by the opposing actions of UFL1 and UFSP1, which governs YAP stability and is indispensable for mammalian kidney development. These findings establish UFMylation as a crucial modulator of Hippo signaling in organogenesis and delineate a previously unrecognized etiological pathway for kidney dysplasia.

## Results

### *Ufl1* deficiency disrupts renal morphogenesis and leads to kidney dysplasia

Our previous work demonstrates that *Ufl1* deficiency induces ER stress and culminates in kidney atrophy in adult mice, indicating UFMylation as a novel regulator of kidney homeostasis (23). However, the mechanistic basis for this phenotype remained to be explored. To determine whether the pathological manifestations arise from a failure in proper organogenesis, we conducted a detailed temporal analysis of kidneys from conditional knockout (CKO) mice. Consistent with our previous findings (23), *Ufl1* deficiency resulted in significant kidney atrophy with a compensatory increase in the number of glomeruli in 1-month-old mice (Fig. S1, *A–C*). Strikingly, this size reduction was already evident at postnatal day 0 (P0) in CKO mice, which exhibit markedly smaller and lighter kidneys compared to the wild-type (WT) mice (Figs. 1, *A–D* and S1, *D* and *E*). Histopathological examination of P0 kidney sections revealed profound architectural disorganization, characterized by a hypoplastic medulla, an expanded renal cortex, and a surge in glomerular number (Fig. 1*E*). Moreover, the glomeruli and tubules in CKO mice displayed pronounced structural aberrations, including collapsed glomeruli and tubules with barely discernible lumens (Fig. 1, *F* and *G*). These observations prompted us to investigate earlier developmental stages. Given that the early renal cortex appears at embryonic day 14 (E14) and the early medullary structure emerges at E18 (39), we analyzed kidneys from WT and CKO embryos at E18.5. Remarkably, the dysplastic phenotype was fully penetrant at E18.5 (Fig. 1*H*), as same as we observed at P0: a hypoplastic medulla, expanded cortex, glomerular hypernumeracy, and severe defects in glomerular and tubular differentiation (Fig. 1, *I* and *J*). Collectively, these results demonstrate that the loss of *Ufl1* disrupts fundamental processes of renal morphogenesis, establishing an essential role for UFMylation in mammalian kidney development.

**Figure 1:**
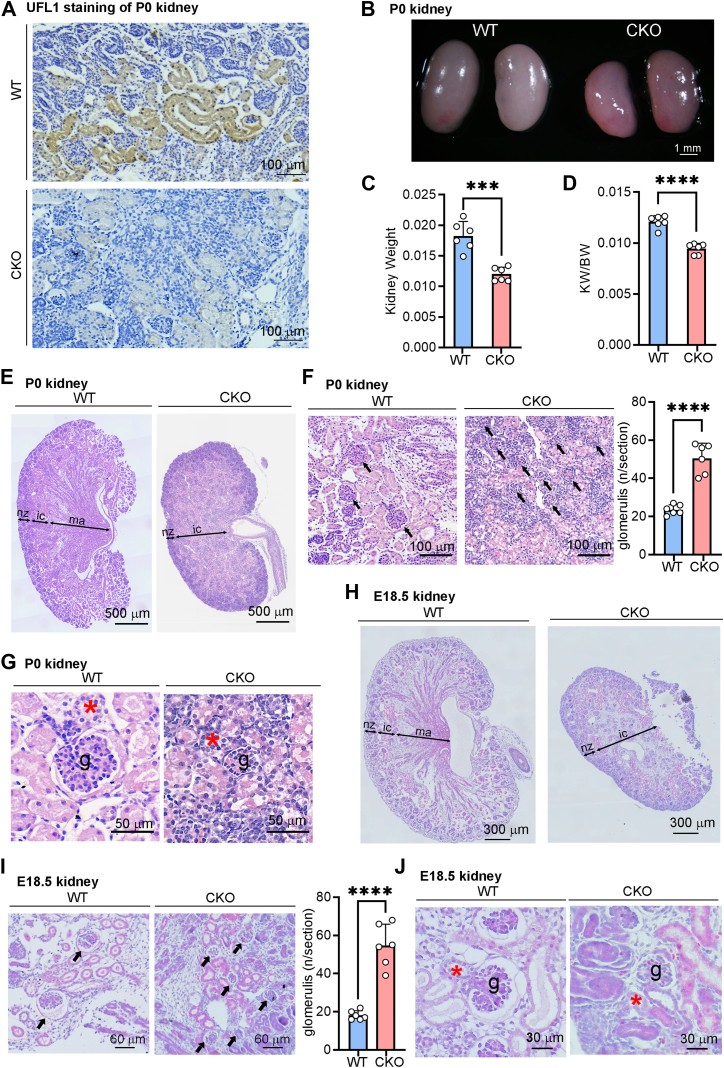
***Ufl1* Deletion results in abnormal kidney development of mice.***A*, kidney *Ufl1* depletion of P0 CKO mice was validated by immunohistochemical staining. Scale bar represent 100 μm. *B*, macroscopic view of WT and CKO mice kidneys at P0. Scale bar represent 1 mm. *C* and *D*, the kidney weight (*C*) and kidney weight to body weight ratio (*D*) of P0 WT and CKO mice was calculated. *E*, H&E staining of kidney sections of P0 WT and CKO mice. *Arrows* point to the papilla. ic: inner cortex; ma: medulla; nz: nephrogenic zone. Scales bar represent 500 μm. *F* and *G*, higher magnification of P0 kidney sections shows glomeruli number (*F*), and shows glomeruli structure and tubules (*G*). g: glomeruli. Scale bars represent 100 μm (*F*), 50 μm (*G*). *Arrows* point to the glomeruli, and the number of P0 glomeruli was counted (*F*). *H*, H&E staining of kidney sections of E18.5 WT and CKO mice. Scales bar represent 300 μm. *I* and *J*, higher magnification of E18.5 kidney sections shows glomeruli numbe (*I*) and shows glomeruli structure and tubules (*J*). Scale bars represent 60 μm (*I*), 30 μm (*J*). *Arrows* point to the glomeruli, and the number of E18.5 glomeruli was counted (*I*).

### *Ufl1* deficiency impairs the YAP-dependent transcriptional program essential for kidney development

Previous report demonstrated that conditional knockout of *Yap* in the renal cap mesenchyme results in early defects in nephron induction, which phenotypic parallels with the *Ufl1* CKO mice models (30). Therefore, we hypothesized that UFMylation might intersect with YAP signaling. By analyzing the Mammalian Organ Development Database (http://evodevoapp.kaessmannlab.org), we found that *YAP*, *UFL1*, and *UFM1* genes exhibit a strikingly congruent expression dynamics (Fig. 2*A*), suggesting a potential co-regulatory relationship. To test this hypothesis, we examined YAP signaling in kidneys from P0 mice, and observed a remarkable accumulation of YAP phosphorylation at S127 and S397 in CKO mice compared to WT littermates (Fig. 2, *B* and *C*), indicating a critical role for UFMylation in regulating YAP activity. Next, we asked whether the observed dysplasia in CKO mice could be attributed to a failure of YAP-dependent transcription. Real-time PCR analysis revealed a profound downregulation of canonical YAP target genes, such as *Areg*, *Myc*, *Ctgf*, and *Cyr61* (40, 41, 42), whereas *Yap* and *Axl* expression were not changed (Fig. 2*D*). Furthmore, a panel of YAP target genes involved in kidney development, including *Tafa5*, *Cited1*, *Pla*_*2*_*g*_*7*_, *Pax8*, *Lim1*, *Sall1* and *Pax2* (43, 44, 45, 46, 47), were also significantly suppressed in CKO kidneys, whereas *Six2* expression remained unchanged (Fig. 2*E*). We speculated that in addition to regulation by YAP, both *Axl* and *Six2* are also modulated by other factors and signaling pathways (48, 49, 50), indicating that YAP-dependent transcription in the kidney is context-dependent and gene-specific. Since YAP directs nephrogenesis primarily through transcriptional regulation (30), we sought to determine whether YAP in turn regulates the UFMylation machinery. Analysis of a public dataset (GSE181400) from *Yap*/*Taz* double-knockout embryonic kidneys showed no significant changes in the expression of most UFMylation components (Fig. S2*A*). In line with this observation, we examined the mRNA and protein expression levels of UFMylation components in renal tubular HK-2 cells by siRNA-mediated YAP depletion, and the results showed that the expression of most UFMylation components were not changed (Fig. S2, *B* and *C*), indicating an unidirectional regulation between UFMylation and YAP signaling. Taken together, these results suggest YAP as a critical downstream effector of UFMylation in renal morphogenesis.

**Figure 2:**
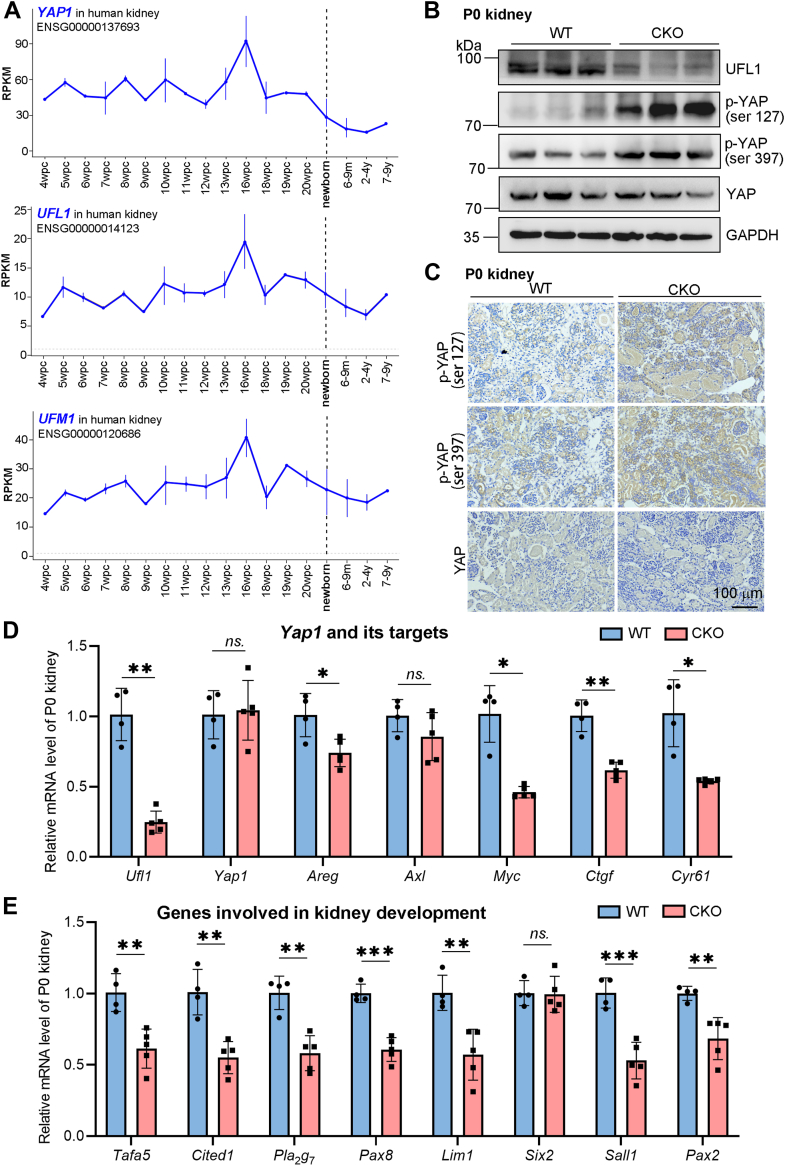
**Loss of *Ufl1* impairs YAP activation in kidney.***A*, gene expression of *YAP*, *UFL1*, and *UFM1* during human kidney development. wpc: weeks post conception. Data sourced from the mammalian developmental biology database, http://evodevoapp.kaessmannlab.org. *B* and *C*, the levels of phospho-YAP (Ser127) and phospho-YAP (Ser397) in kidney of P0 mice were detected by western blotting (*B*) and immunohistochemical staining (*C*). Scale bar represent 100 μm. *D* and *E*, the mRNA levels of YAP-dependent genes (*Areg*, *Axl*, *Myc*, *Ctgf*, and *Cyr61*) (*D*) and genes (*Tafa5*, *Cited1*, *Pla*_*2*_*g*_*7*_, *Pax8*, *Lim1*, *Six2*, *Sall1*, and *Pax2*) involved in kidney development (*E*) in kidney of P0 mice were analyzed by RT–qPCR.

### UFL1 controls YAP stability, localization, and transcriptional activation

To delineate the detailed mechanisms by which UFL1 regulates YAP, we extended our analysis in HK-2 cells. Consistent with our *in vivo* observations, UFL1 depletion markedly enhanced YAP phosphorylation at S127 and S397 (Fig. 3*A*). This effect was also observed in 3T3-L1 preadipocytes, the human normal colonic epithelial cells NCM460, and human embryonic pluripotent stem cells (HESCs) (Fig. S3, *A–C*), supporting the evolutionary conservation of this regulatory axis. Although phosphorylation of YAP at S127 and S397 is mediated by the activated (phosphorylated) forms of LATS1/2, our results revealed that UFL1 depletion did not affect the phosphorylation status of LATS1/2 or its cofactor MOB kinase activator 1 (MOB1) (Figs. 3*A* and S3, *A–C*). Instead, loss of UFL1 markedly enhanced the interaction between YAP and LATS1/2 (Fig. 3*B*). We hypothesized that UFL1 acts to restrain the Hippo kinase cascade, thereby preventing the inactivation of YAP *via* two coordinated mechanisms: cytoplasmic sequestration and proteasomal degradation as schematized (Fig. 3*C*). To support this model, we found that UFL1 depletion significantly attenuated the interaction between YAP and its key nuclear partner TEADs (Fig. 3*D*), indicating a loss of YAP transcriptional activity. Concomitantly, we observed enhanced binding of YAP to both 14-3-3 and the SCF^β-TrCP^ ligase (Fig. 3*E*), coupled with a pronounced increase in YAP ubiquitination (Fig. 3*F*). To directly assess the impact on protein stability, we examined YAP half-life in cells following cycloheximide (CHX) treatment and found that UFL1 depletion accelerated YAP degradation (Fig. 3*G*).

**Figure 3:**
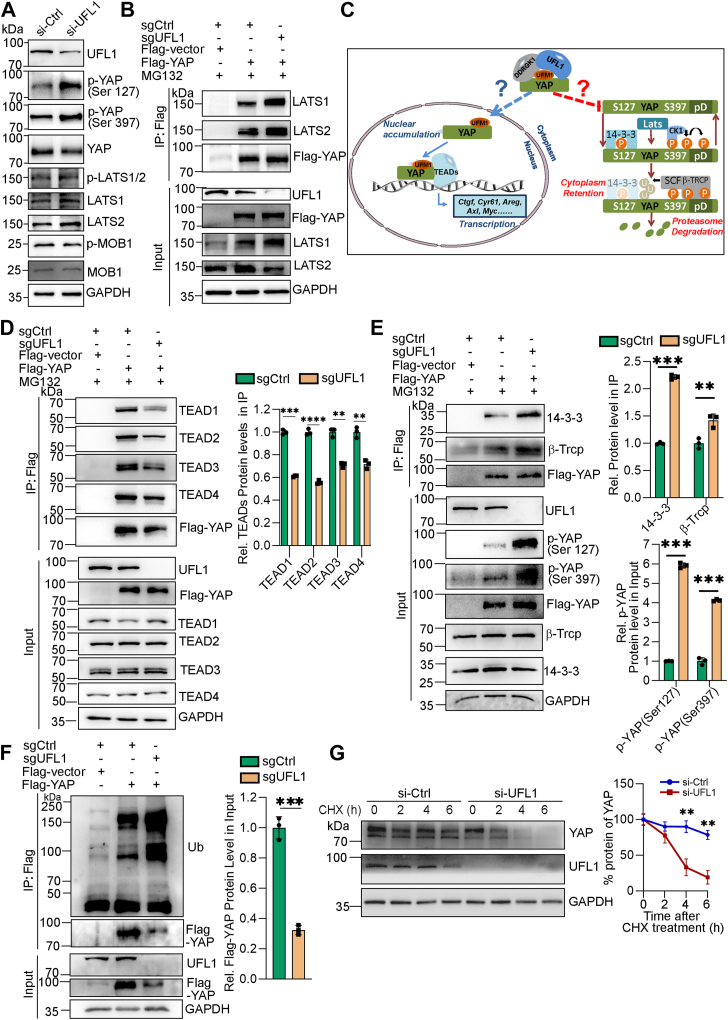
**Deletion of *UFL1* enhances YAP phosphorylation and ubiquitination.***A*, Western blot analysis of phospho-YAP (Ser127), phospho-YAP (Ser397), phospho-LATS1/2 and phospho-MOB1 levels in HK-2 cells with depleted of *UFL1* by siRNAs. *B*, the association of LATS1/2 with YAP was analyzed by immunoprecipitation followed by Western blot analysis in HEK-293T cells with *UFL1* depletion in the presence of MG132 (20 μm, 8 h). *C*, hypothesized model, UFL1 prevents the inactivation of YAP *via* two coordinated mechanisms: cytoplasmic sequestration and proteasomal degradation. *D and E*, the association of TEADs (*D*), 14-3-3, and β-TrCP (*E*) with YAP was analyzed by immunoprecipitation followed by Western blot analysis in HEK-293T cells with *UFL1* depletion in the presence of MG132 (20 μm, 8 h). The TEADs protein levels in *IP* were analysis (*right panel* of Fig. *D*). The 14-3-3 and β-TrCP protein levels in *IP*, and phospho-YAP (Ser127) and phospho-YAP (Ser397) levels in *Input* were analysis (*right panel* of Fig. *E*). *F*, ubiquitination of YAP was analyzed by immunoprecipitation followed by Western blot analysis in HEK-293T cells with *UFL1* depletion. Flag-YAP protein level in *Input* was analysis (*right panel*). *G*, YAP stability in HK-2 cells with *UFL1* deletion was examined by western blotting. The cells were treated with 100 μg/ml CHX for the indicated times. The graph represents quantification of the YAP protein levels (*right panel*).

As nuclear access is critical for YAP transcriptional activity (51), we analyzed the effect of UFL1 on its cellular distribution. Immunofluorescence analysis revealed that UFL1 deficiency caused a pronounced redistribution of endogenous YAP from the nucleus to the cytoplasm (Fig. 4*A* and S4*A*), and consequently, was evidenced by substantial downregulation of canonical YAP target genes (Fig. 4*B*). In addition, MTT and colony formation assays showed that loss of UFL1 impaired cellular growth and proliferation (Fig. 4, *C* and *D*, and S4*B*), which is in accordance with loss of YAP transcriptional activity. Together, these results revealed that UFL1 is a critical upstream suppressor of the Hippo pathway by shielding YAP from phosphorylation and proteasomal degradation; therefore, UFL1 ensures YAP nuclear localization and promotes its transcriptional activity essential for cellular proliferation and renal development.

**Figure 4:**
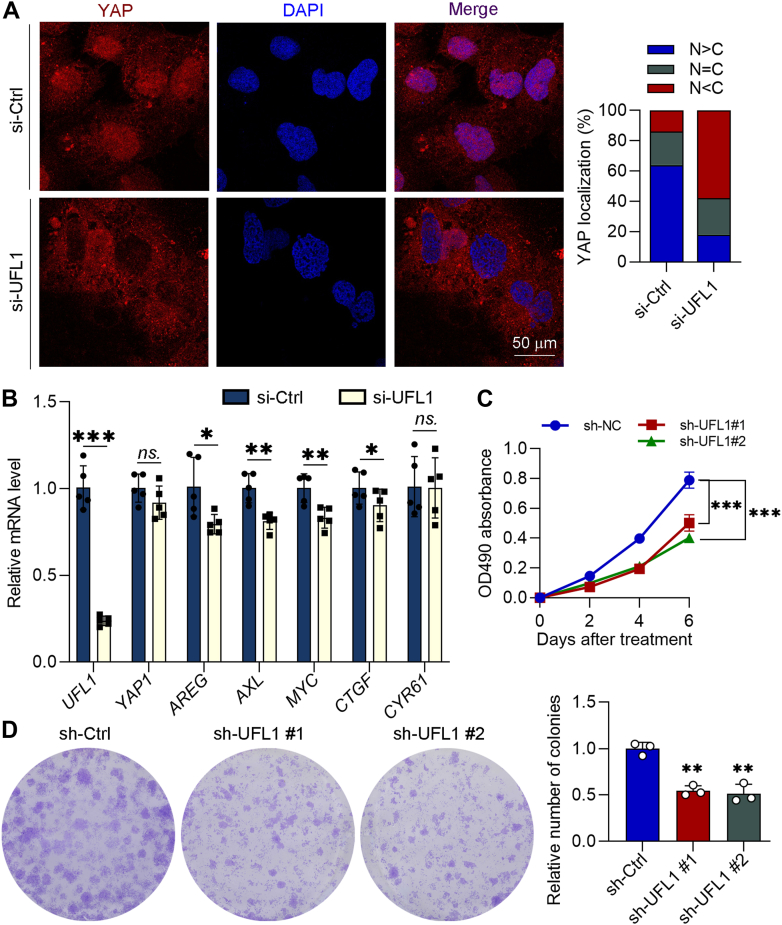
**Loss of *UFL1* impairs YAP localization and transcriptional function affecting cell growth and proliferation.***A*, immunofluorescent staining of YAP in HK-2 cells with *UFL1* deficiency. Percentage of cells with the relative ratio of nucleo-YAP *vs*. cytoplasmic-YAP was counted (*right panel*). Scale bar represent 50 μm. *B*, the mRNA levels of YAP-dependent genes (*AREG*, *AXL*, *MYC*, *CTGF*, and *CYR61*) in HK-2 cells with *UFL1* deficiency were analyzed by RT–qPCR. *C* and *D*, the MTT assay (*C*) and colony formation assay (*D*) of HK-2 cells with *UFL1* depletion. The number of colonies were counted (*right panel* of Fig. *D*).

### YAP is a *bona fide* substrate for UFMylation at its K76 residue

To determine whether YAP is a direct target for UFMylation, we firstly examined the interaction between YAP and UFL1, and the co-immunoprecipitation results showed that YAP binds with UFL1 in HEK293T cells (Fig. 5*A*). We next asked whether YAP undergoes UFMylation. The UFMylation assay was performed in cells by co-expressing YAP with the UFMylation components UBA5, UFC1, UFL1, UFM1, and DDRGK1, and the results revealed that an active form of UFM1 with an exposed carboxy (C)-terminal glycine-83 residue (UFM1-ΔC2), but not an inactive form of UFM1 lacking the C-terminal glycine-83 residue (UFM1-ΔC3), could conjugate to YAP (Fig. 5*B*). Importantly, the *in vitro* UFMylation assays confirmed that YAP is a *bona fide* substrate for direct UFMylation (Fig. 5*C*). To identify the specific UFMylation sites on YAP, we performed immunoprecipitation of YAP followed by mass spectrometry, which identified K76 as an UFMylation site (Fig. 5*D*). K76 residue is located upstream of the transcriptional activation domain and is evolutionarily conserved across species (Fig. 5*E*). To further confirm this UFMylation residue, we generated a lysine-to-arginine point mutation (K76R), and the UFMyaltion assay results showed that the K76R mutation nearly abolished YAP UFMylation *in vivo* (Fig. 5*F*). In summary, these findings demonstrated that YAP is a *bona fide* substrate for UFMylation at its K76 residue.

**Figure 5:**
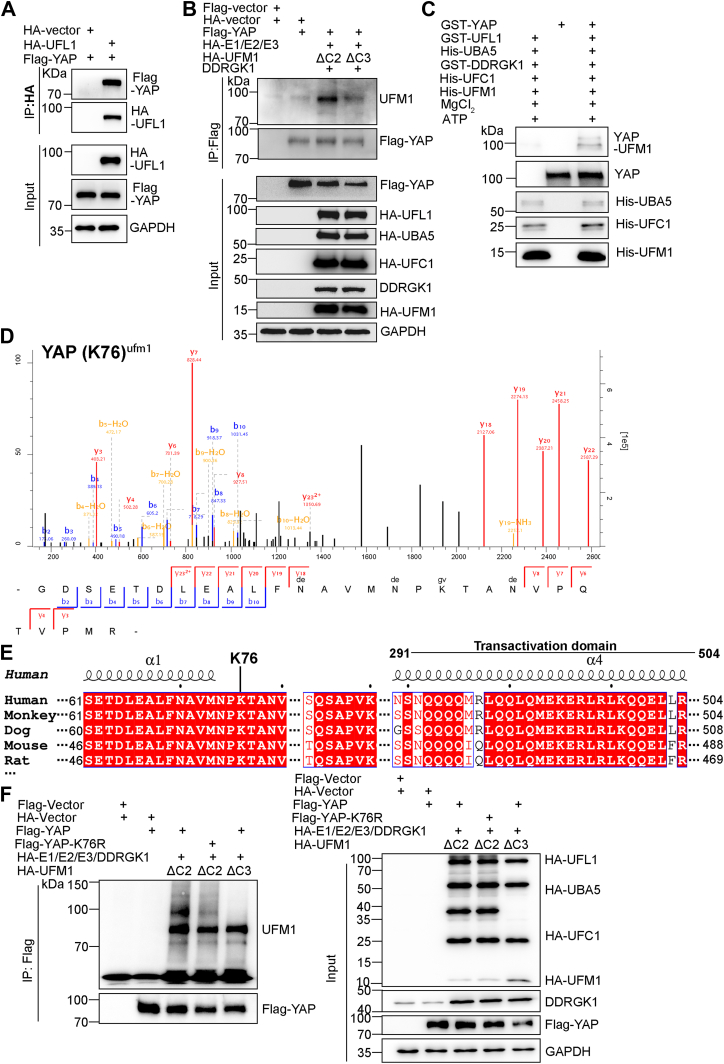
**YAP is a *bona fide* substrate for UFMylation at K76.***A*, Western blot analysis of the mutual interaction between UFL1 and YAP in HEK-293T cells by co-immunoprecipitation with anti-HA magnetic beads. *B*, after expressing the UFMylation components, UFMylation of YAP in HEK-293T cells was analysed by western blotting. *C*, YAP UFMylation *in vitro* was detected by western blotting with anti-UFM1 antibody. *D* and *E*, YAP UFMylation at the K76 site was detected by mass spectrometry (*D*), and the site is located upstream of the transactivation domain and is conserved across multiple species (*E*). *F*, UFMylation assay of the YAP-WT and its mutant (YAP-K76R) in HEK-293T cells.

### UFSP1 serves as the dominant de-UFMylating protease for YAP

Recent studies have reported that UFSP1 is also an active protease as UFSP2 which regulates UFM1 maturation and substrates de-UFMylation (6, 8), therefore we determine the primary protease responsible for YAP de-UFMylation. The *in vivo* UFMylation assays revealed that loss of UFSP1, but not UFSP2, markedly enhanced YAP UFMylation, identifying UFSP1 as the principal de-UFMylating enzyme for YAP in cells (Fig. 6, *A* and *B*). Intriguingly, *in vitro* assays showed that both UFSP1 and UFSP2 could cleave the UFM1-YAP conjugates (Figs. 6*C* and S5*A*), likely because the UFM1-ΔC2 substrate used was already mature (1), bypassing the processing requirement. Strikingly, we observed a functional divergence between these proteases in cellular contexts. Knockdown of UFSP1 suppressed YAP phosphorylation, thereby enhancing its stability and activating downstream targets including *AREG*, *AXL*, *MYC*, *CTGF*, and *CYR61* (Fig. 6, *D* and *E*). Conversely, Knockdown of UFSP2 increased YAP phosphorylation and degradation, leading to inhibition of its target genes (Fig. S5, *B* and *C*). Exogenous YAP exhibited pronounced protein accumulation in UFSP1-deficient cells compared to UFSP2-deficient cells (Fig. 6*F*). Consistently, the half-life of YAP was extended upon UFSP1 deficiency, but not UFSP2 deficiency (Figs. 6*G* and S5D). Furthermore, induction of canonical YAP targets (*CTGF* and *CYR61*) by exogenous YAP was potentiated in UFSP1-deficient cells but abolished in UFSP2-deficient cells (Fig. 6*H*). Taken together, we have identified UFSP1 as the dominant de-UFMylating protease for YAP, distinguished from UFSP2 both in substrate specificity and functional outcome, indicating a previously unrecognized regulation in UFMylation system.

**Figure 6:**
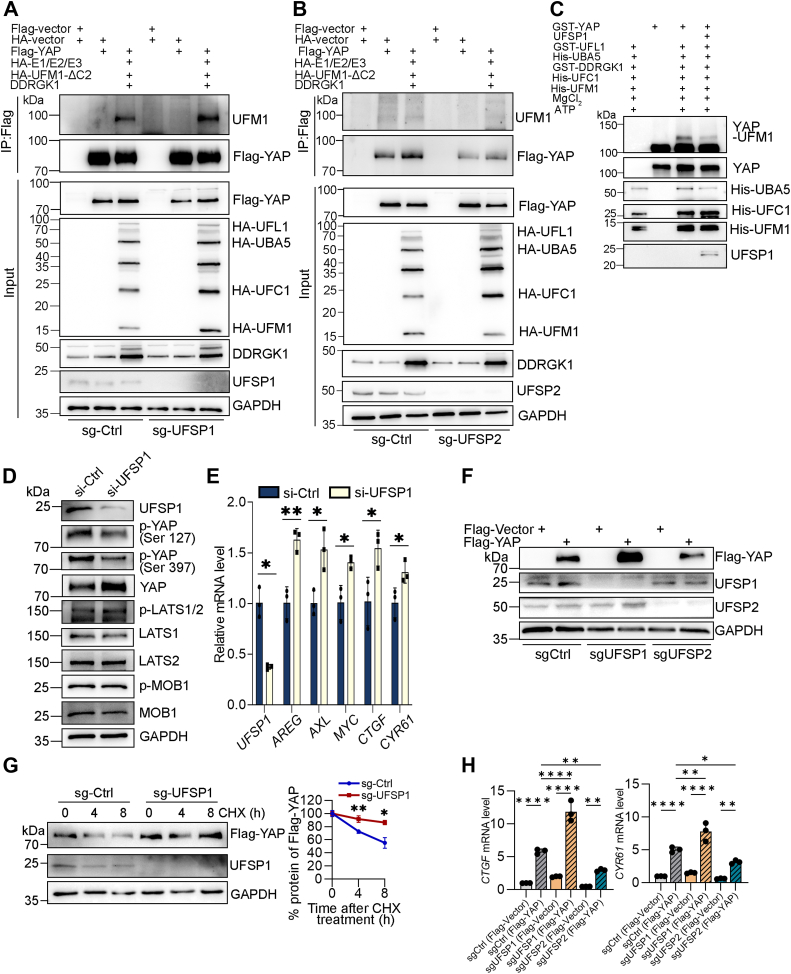
**UFSP1 serves as the dominant de-UFMylating protease for YAP.***A* and *B*, after expressing the UFMylation components, YAP UFMylation in HEK-293T cells with UFSP1 (A) or UFSP2 (*B*) depletion was analysed by western blotting with anti-UFM1 antibody. *C*, the effect of UFSP1 on YAP UFMylation *in vitro* was analysed by western blotting. *D*, the phospho-YAP (Ser127), phospho-YAP (Ser397), phospho-LATS1/2 and phospho-MOB1 levels in HK-2 cells with loss of UFSP1 were detected by western blotting. *E*, the mRNA levels of YAP targets (*AREG*, *AXL*, *MYC*, *CTGF*, and *CYR61*) in UFSP1 deletion HK-2 cells were analyzed by RT–qPCR. *F*, Western blot analysis of Flag-YAP level in HEK-293T cells with loss of UFSP1 or UFSP2. *G*, Flag-YAP stability in HEK-293T cells with deletion of UFSP1 was examined by western blotting. The cells were treated with 100 μg/ml CHX for the indicated times. The graph represents quantification of the Flag-YAP protein levels (*right panel*). H, RT-qPCR analysis of *CTGF* and *CYR61* mRNA levels induced by Flag-YAP in HEK-293T cells with loss of UFSP1.

### K76 residue is essential for UFMylation-dependent YAP activation

To further determine whether UFMylation at K76 regulates YAP activity and function, we firstly investigated the functional consequence by expressing Flag-YAP WT and K76R mutant in HK-2 cells. The results showed that the K76R mutant exhibited higher levels of YAP phosphorylation and stronger association with LATS1/2 compared with YAP-WT (Fig. 7, *A* and *B*), which correlates with its predominantly cytoplasmic localization, in contrast to the nuclear enrichment of YAP-WT (Fig. 7*C*). Accordingly, the YAP-WT potently induced the expression of canonical target genes, including *AREG*, *CTGF*, *MYC* and *AXL*, while the K76R mutant failed (Figs. 7*D* and S6*A*). Furthermore, the K76R mutant exhibited a significantly shorter protein half-life compared to YAP-WT (Fig. 7*E*), indicating that UFMylation is critical for YAP stability. Next, we evaluated whether K76 in YAP is critical for UFMylation-mediated YAP regulation. As expected, loss of UFSP1 markedly enhanced the nuclear accumulation of YAP-WT (Figs. 7*F* and S6*B*). Conversely, the K76R mutant completely abrogated this effect, reverting YAP to a cytoplasmic localization despite UFSP1 depletion (Figs. 7*F* and S6*B*). In addition, reintroducing K76R mutant into UFSP1-knockdown cells did not restore the ability of UFMylation mediated YAP transcriptional activation (Fig. 7*G*). Colony formation assays further revealed that UFSP1 knockdown enhanced cellular growth in a YAP-dependent manner, whereas this pro-proliferative effect was entirely abolished in cells expressing YAP-K76R mutant (Figs. 7*H* and S6*C*). Taken together, we demonstrated that UFMylation at K76 of YAP is essential for its nuclear localization, transcriptional activation and cell proliferation, indicating a functional regulation of YAP UFMylation in kidney development.

**Figure 7:**
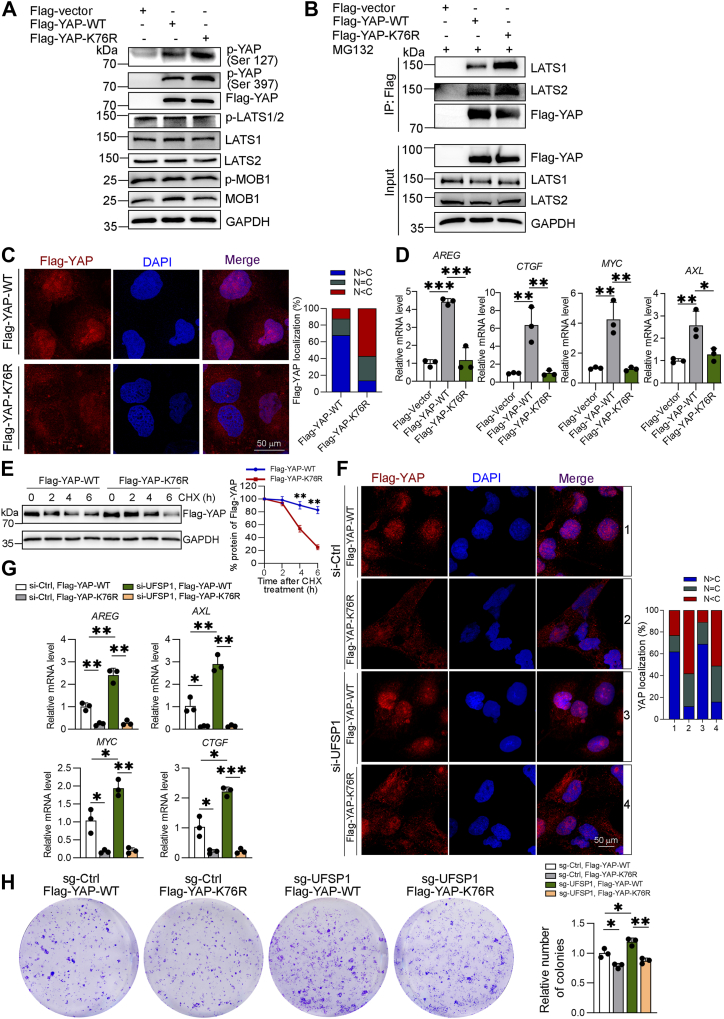
**K76 residue is essential for YAP activation and cellular proliferation.***A*, Western blot analysis of phospho-YAP (Ser127), phospho-YAP (Ser397), phospho-LATS1/2 and phospho-MOB1 levels in HK-2 cells with Flag-YAP-WT or Flag-YAP-K76R overexpression in the presence of MG132 (20 μm, 8 h). *B*, the association of LATS1/2 with Flag-YAP-WT or Flag-YAP-K76R was analysed by immunoprecipitation followed by Western blot analysis in HEK-293T cells in the presence of MG132 (20 μm, 8 h). *C*, immunofluorescent staining of Flag-YAP-WT or Flag-YAP-K76R in HK-2 cells. Percentage of cells with the relative ratio of nucleo-Flag-YAP *vs*. cytoplasmic-Flag-YAP was counted (*right panel*). Scale bar represent 50 μm. *D*, RT-qPCR analysis of YAP targets expression induced by Flag-YAP-WT or Flag-YAP-K76R (*AREG*, *CTGF*, *MYC*, and *AXL*) in HK-2 cells. E, Flag-YAP-WT or Flag-YAP-K76R stability was examined by western blotting in HK-2 cells. The cells were treated with 100 μg ml^−1^ CHX for the indicated times. The graph represents quantification of the Flag-YAP protein levels. *F*, immunofluorescent staining of Flag-YAP-WT or Flag-YAP-K76R in HK-2 cells with deletion of UFSP1 by siRNAs. Percentage of cells with the relative ratio of nucleo-Flag-YAP *vs*. cytoplasmic-Flag-YAP was counted (*right panel*). Scale bar represent 50 μm. *G*, RT-qPCR analysis of YAP targets expression induced by Flag-YAP-WT or Flag-YAP-K76R (*AREG*, *CTGF*, *MYC*, and *AXL*) in HK-2 cells with deletion of UFSP1 by siRNAs. *H*, the colony formation assay of UFSP1 deletion HEK-293T cells expressing Flag-YAP-WT or Flag-YAP-K76R. The number of colonies were counted (*right panel*).

## Discussion

In this study, we delineate a previously unrecognized regulatory axis in which UFMylation acts as a critical post-translational switch controlling the Hippo pathway effector YAP to ensure proper mammalian kidney development. We demonstrate that UFMylation modulates the YAP-LATS1/2 interaction and promotes the UFMylation ligase UFL1 directly binds with YAP and catalyzes UFM1 conjugation at its K76 residue, which stabilizes YAP by antagonizing the canonical Hippo kinase cascade, thereby shielding YAP from 14-3-3 mediated cytoplasmic sequestration and SCF^β-TrCP^ dependent proteasomal degradation. Consequently, UFMylation licenses the nuclear accumulation of YAP and the activation of a transcriptional program essential for renal morphogenesis. Additionally, we identify UFSP1, rather than the widely recognized protease UFSP2 (4, 7, 10, 52, 53), as the dominant de-UFMylating enzyme for YAP, unveiling a finely tuned UFMylation cycle that dynamically governs YAP activity (Fig. 8). Collectively, our work expands the physiological scope of UFMylation beyond endoplasmic reticulum homeostasis to include direct regulation of a central developmental signaling.

**Figure 8:**
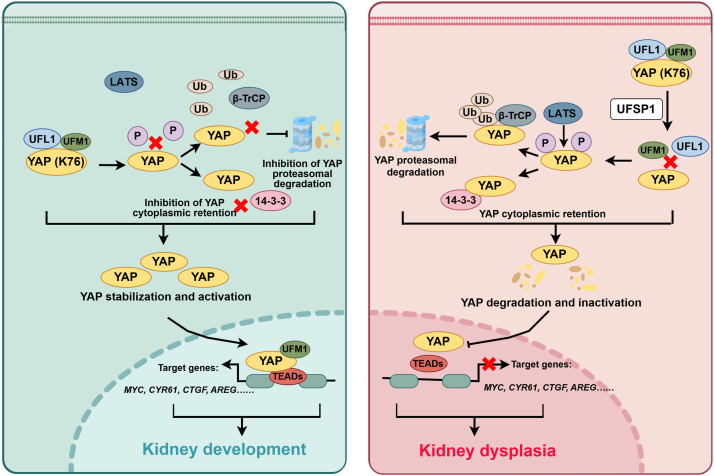
**Graphical Abstract.** UFMylation of YAP at K76 protects YAP from 14-3-3 mediated cytoplasmic sequestration and SCF^β-TrCP^ dependent proteasomal degradation, thereby promoting the nuclear accumulation of YAP and the activation of a transcriptional program essential for renal morphogenesis. UFSP1 is identified as the dominant de-UFMylating enzyme for YAP that controls its activity.

Recent studies have demonstrated that UFMylation is essential for murine embryogenesis, hematopoiesis and tissue homeostasis (54, 55, 56, 57, 58). Depletion of UFL1 resulted in intestinal and liver hypoplasia and dysfunction by disrupting endoplasmic reticulum homeostasis and ribosome homeostasis respectively (56, 57), indicating UFMylation serves as an indispensable safeguard for development. Notably, our findings position UFMylation as a direct upstream regulator in the Hippo-YAP signaling network. The Hippo pathway is renowned as a central regulator of organ size (26, 59, 60), but the upstream signals that modulate its activity in specific developmental contexts remain incompletely understood. Here, we show that UFMylation acts as a fundamental brake on the Hippo kinase cascade, effectively setting a permissive threshold for YAP activation. This mechanism is distinct from our previously characterized role of UFMylation in endoplasmic reticulum stress management (23), highlighting a novel function for this modification in transcriptional regulation and organ development.

Although YAP is strongly implicated by our phenotypic and mechanistic findings, we note that UFMylation also targets additional substrates, which may contribute to the full spectrum of renal defects arising from *Ufl1* ablation. In principle, a global transcriptomic comparison between *Ufl1* CKO and *Yap* CKO mice would provide a quantitative estimate of the extent to which *Ufl1* loss phenocopies YAP-dependent transcriptional programs and would help disentangle YAP-dependent from YAP-independent UFMylation outputs. However, because tissues from *Yap* CKO mice were unavailable for the present study, we could not perform this comparative analysis. Notwithstanding this limitation, the severe renal dysplasia caused by *Ufl1* deletion closely phenocopies the loss of *Yap* activity, suggesting that YAP may be a major contributor to the renal pathogenesis driven by *Ufl1* deficiency. Accordingly, UFMylation might serve as a tissue-specific rheostat for Hippo signaling, with potential broader implications for other YAP-dependent developmental and regenerative processes.

A particularly intriguing finding in the present study is the marked functional divergence between the two de-UFMylating proteases UFSP1 and UFSP2 (7). Although both enzymes can cleave UFM1 from YAP *in vitro*, we demonstrate YAP might be the first identified physiological substrate preferentially regulated by UFSP1 in cells. UFSP1 deficiency promotes YAP stabilization and activation, whereas UFSP2 deficiency promotes YAP degradation in this cellular context, indicating UFSP2 may primarily facilitate UFM1 maturation or alternatively participate in regulating other E3 ligases or upstream components that indirectly suppress YAP activity. These findings illuminate a sophisticated regulatory architecture in which UFSP1 and UFSP2 exert complementary yet distinct roles to ensure precise control of YAP stability and activity.

The UFMylation-YAP regulatory axis uncovered here also holds broad therapeutic implications. Given the well-established roles of YAP hyperactivation in cancer (61, 62) and tissue regeneration (63, 64, 65), targeting this pathway offers attractive translational opportunities. The substrate specificity for UFSP1 toward YAP challenges the conventional view of redundant protease function and opens new avenues for therapeutic intervention, as targeting UFSP1 could offer a selective modulation of YAP activity without globally disrupting UFMylation system, providing a promising strategy for treating YAP-driven pathologies, from developmental malformations to cancer and regenerative disorders.

## Experimental procedures

### Reagents

All antibodies, buffers, and other reagents used throughout this work are described in Table S1.

### Mice

All mice were bred on a mixed genetic background. *Pax8*-Cre transgenic mice (C57BL/6J) were bred with mice carrying loxP-flanked *Ufl1* (*Ufl1*^flox/flox^) allele to generate nephron tubule-specific *Ufl1*-knockout model as previously described (23). All mice were housed, maintained, and used according to (including the mice euthanasia procedure) the regulations and guidelines of the Animal Care and Use Committee of Hangzhou Normal University, and approved by the Animal Care and Use Committee of Hangzhou Normal University (Approval No. 2023190). Mice were housed in a climate-controlled, specific-pathogen-free animal facility at 22 to 24 °C with ambient humidity kept within a 40 to 60% range, with a 12:12 h light-dark cycle and ad libitum access to chow (1,010,085, Synergy Biology) and water. Timed matings were generated, with day of plug counted as embryonic day (E) 0.5. Pregnant females were euthanized at E18.5. Embryonic mice with the desired genotype were randomly selected, with Cre-negative littermates used as controls. Embryonic kidneys were quickly dissected and preserved in formalin or snap-frozen in liquid nitrogen and stored at −80 °C until further analysis.

### Cells

HK-2 cells were maintained in MEM, HEK-293T and 3T3-L1 cells in DMEM, NCM460 cells in RPMI-1640, and HESCs in DMEM/F12 (supplemented with 10% FBS and 1% penicillin-streptomycin). Unless specified differently, media was changed every other day and cells were maintained at 37 °C in 5% CO_2_. For reduction of UFL1, UFSP1, UFSP2, or YAP gene expression, HK-2 cells were transfected by siRNAs. The siRNAs were a mixture of two siRNAs, and these sequences are provided in Table S2. HK-2 cells (∼30% confluence) in a 6-well plate were transfected with UFL1, UFSP1, UFSP2 or YAP siRNAs (an equal amount of each was mixed) and scrambled siRNA using Lipofectamine 6000. After 48 h transfection, total RNA was extracted from cells for RT–qPCR analysis. After 72 h transfection, protein level was detected by Western blot analysis. UFL1, UFSP1 or UFSP2 gene knockout in HEK-293T cells was achieved using a CRISPR-Cas9 system. sgRNA was designed to complement a 20-nucleotide sequence within an exon of the target UFL1, UFSP1, or UFSP2 gene, directly upstream of a 5′-NGG-3′ protospacer adjacent motif, and these sgRNA sequences are provided in Table S3. The Cas9 nuclease, complexed with the sgRNA, was introduced into the HEK-293T cells *via* lipid-based transfection. Frameshift mutations introduced by these indels were confirmed by DNA sequencing of the target locus. Following confirmation of successful knockout *via* Western blot analysis, the cells were used in subsequent experiments.

### Histology and immunohistochemistry

Isolated kidney tissues were fixed in 4% formalin fixative for 48 h at 4 °C. After dehydration, tissues were embedded in paraffin and sliced to a thickness of 5 μm. The sections were deparaffinized, rehydrated, stained with H&E following the standard protocol. For immunohistochemistry, slides were deparaffinized, rehydrated and incubated in PBS containing 0.1% Triton X-100 for 10 min followed by wash with PBS (3 × 5 min). The endogenous peroxidase activity was blocked by incubating in 3% H_2_O_2_ solution for 10 min. After three washes with PBS, the sections were heated in a sodium citrate solution (0.01 M, pH 6.0) for 20 min in a 95 °C water bath to retrieve antigen and then blocked with 3% BSA (25 °C, 1 h) to suppress nonspecific background staining. The sections were incubated with anti-UFL1 Rabbit pAb, anti-Phospho-YAP (Ser127) Rabbit mAb, anti-Phospho-YAP1 (Ser397) Rabbit pAb or anti-YAP Rabbit pAb (1:1000 dilution) overnight at 4 °C. IHC detection kit and DAB Horseradish Peroxidase Color Development Kit were used for visualization of the binding of antibody. Counterstaining was performed with hematoxylin.

### Plasmid construction and transfection

UBA5, UFC1, UFL1, and UFM1 (UFM1ΔC2 or UFM1ΔC3) cDNAs were cloned into pSG5-HA-Vector. DDRGK1 cDNA was cloned into pcDNA3.1-Vector. YAP cDNA was cloned into the pcDNA3.0 to 3 × Flag-6 × His-Vector, and the lysine (76)-to-arginine mutation in YAP was generated by site-directed mutagenesis (the primer: 5′-GCACGTTGGCCGTCCTGGGGTTCATGACG-3′ and 5′-CGTCATGAACCCCAGGACGGCCAACGTGC-3′). The shUFL1 sequences were cloned into the LV10-U6 lentivirus expression vector for stable cell line generation. Bacteria expressing GST-tagged UFL1, DDRGK1, and YAP were generated using the pGEX-6P-1 system. Plasmid transfection was performed with Lipofectamine 6000, according to the manufacturer’s instructions.

### Immunofluorescence analysis

Cells were fixed using 4% formalin fixative and immersed in PBS. Antigen retrieval was performed with sodium citrate solution (0.01 M, pH 6.0) and permeabilized in 0.5% Triton X-100 for 10 min. Cells were then blocked with 3% BSA for 2 h at room temperature. The cells were incubated overnight at 4 °C with primary antibodies diluted (1:500) in PBS containing 0.2% TritonX-100. The cells were washed three times for 10 min with PBS, and immune complexes were detected by incubation for 2 h at room temperature with Rhodamine (TRITC)-conjugated Goat Anti-Rabbit IgG at a dilution of 1:500 in PBS. The cells were then washed three times for 10 min with PBS, exposed to DAPI for 5 min to stain nuclei, washed briefly with PBS, mounted with antifade reagent and sealed with a cover glass, followed by observation and photograph with a laser scanning confocal microscopy.

### Stable cell line generation and colony formation assay

To harvest lentivirus containing shRNA against UFL1, HEK-293T cells were transfected with shUFL1-expressing plasmids (LV10-U6), together with psPAX2 and pMD2.G. After 48 h and 72 h transfection, the culture medium was collected to harvest virus particles. HK-2 cells were infected with lentivirus containing shUFL1, and stable cell lines were generated by puromycin (1 μg/ml) selection. HK-2 cells were seeded into 12-well plates at a density of 2000 cells per well, and cultured in complete medium for 7 days, followed by staining of 0.1% crystal violet solution. Stained colonies were imaged, and the relative colony intensity was quantitated using the ImageJ Image processing program.

### Assays for UFMylation *in vitro* and *in vivo*

For the *in vitro* UFMylation assay, GST-tagged YAP, UFL1 and DDRGK1 were ectopically expressed in BL21 cells and purified using Glutathione Beads. Recombinant human His-UBA5, His-UFC1, His-UFM1 proteins were obtained from R&D Systems. His-UBA5 (0.1 μm), His-UFC1 (0.1 μm), His-UFM1 (0.1 μm), GST-UFL1 (0.1 μm), GST-DDRGK1 (0.1 μm) and GST-YAP (0.1 μm) were mixed in a reaction buffer (0.05% BSA and 50 mM HEPES (pH 7.5)) containing 5 mM ATP and 10 mM MgCl_2_ and incubated at 30 °C for 2 h. The mixtures were boiled in SDS sample buffer containing 5% mercaptoethanol for 10 min, followed by Western blot analysis.

For the *in vivo* UFMylation assay, HEK-293T cells were harvested after being transfected with the appropriate constructs for 36 h. Cells were lysed by boiling in buffer (150 mM Tris-HCl (pH 8.0), 5% SDS, and 30% glycerol) for 10 min. Cell lysates were diluted 20-fold with buffer A (50 mM Tris-HCl (pH 8.0), 150 mM NaCl, 0.5% NP-40 and 2 mM N-ethylmaleimide) and protease inhibitor Cocktail. After incubation with anti-Flag Affinity Gel overnight at 4 °C, the immunoprecipitates were subjected to SDS-PAGE followed by Western blot analysis.

### Western blot analysis

Total protein of kidney tissues or cells was extracted by RIPA buffer supplemented with protease inhibitor Cocktail, separated by SDS-PAGE, and transferred to PVDF membranes. The membranes were blocked in 5% milk and stained with the appropriate primary antibodies (1:1000 dilution) and secondary antibodies (1:10,000 dilution). After washing with TBST (Tris-Buffered Saline with Tween), the membranes were scanned and analyzed.

### RT-qPCR analysis

Total RNA was isolated from tissues or cells with the use of TRIzol. RNA was subjected to reverse transcription with the use of the HiScript II Q RT SuperMix, and the resulting cDNA was subjected to real-time PCR analysis with the ChamQ Blue Universal SYBR Master Mix in a CFX96 Touch Real-Time PCR Detection System (Bio-Rad Laboratories). Relative gene expression was calculated by the ΔΔCT method and was normalized by β-actin gene expression. PCR primer sequences are provided in Table S4 and S5.

### Mass spectrometry

HEK293T cells were transfected with Flag-tagged YAP, DDRGK1, as well as HA-UBA5/UFC1/UFL1/UFM1ΔC2 for 36 h. The cells were lysed with lysis buffer (50 mM Tris-HCl pH 7.5, 150 mM NaCl, 1 mM EDTA, 0.5% NP-40). Flag-YAP was immunoprecipitated by incubation with anti-FLAG Affinity Gel overnight at 4 °C. Flag-YAP immunoprecipites were resolved by SDS-PAGE and gel pieces were digested with trypsin at 37 °C overnight. The tryptic peptides were subjected to an EASY-nLC 1000 UPLC system followed by tandem mass spectrometry (MS/MS) in an Orbitrap Fusion Mass Spectrometer (Thermo Scientific). The resulting MS/MS data were processed using Maxquant (1.5.2.8) and performed by Micrometer Biotech Company (Hangzhou, China), and the ufmylation sites of YAP was identified.

### Quantification and statistical analysis

Data are expressed as the mean ± SD of at least three independent experiments for each experimental group. Student’s *t* test was used for comparisons between two groups, one-way analysis of variance followed by Tukey corrected *post hoc t* test was used for multi-group comparisons. A value of *p* < 0.05 was considered statistically significant. ∗*p* < 0.05, ∗∗*p* < 0.01, ∗∗∗*p* < 0.001, and ∗∗∗∗*p* < 0.0001; *ns*, no significance.

### Data availability

Requests for further information, resources, and reagents should be directed to and will be fulfilled by the lead contact, Jiang Liu (liujiang1120@hznu.edu.cn).

## Supporting information

This article contains supporting information.

## Conflict of interest

The authors declare that they have no conflicts of interest with the contents of this article.
