# Supplementary material for: UFMylation orchestrates kidney development through YAP stabilization
Source: J Biol Chem. 2026 Jun 22;302(9):113285. doi: 10.1016/j.jbc.2026.113285 (PMC13476621; doi:10.1016/j.jbc.2026.113285)
Supplement: Supplementary file 1 [file mmc1.docx]

**Supporting information**

**UFMylation orchestrates kidney development through YAP stabilization**

Yi Chen^1^, Longyi Wan^1^, Wei Chen^1^, You Zhou^1^, Fuzhao Zhang^1^, Junyan Zhang^1^, Chenlu Zhang^1^, Qian Liang^1^, Yu Jiang^1^, Ying Wang^1^, Yajun Duan^2^, Yu-Sheng Cong^1^, Jiang Liu^1^*

From the ^1^Zhejiang Key Laboratory of Medical Epigenetics, Department of Cell Biology and Genetics, School of Basic Medical Sciences, Hangzhou Normal University, Hangzhou, Zhejiang, China; ^2^Department of Cardiology, The First Affiliated Hospital of USTC, Division of Life Sciences and Medicine, University of Science and Technology of China, Hefei, Anhui, China

**Figure S1.** ***Ufl1* deficiency results in kidney atrophy in 1-month-old mice.** A, macroscopic view of WT and CKO kidneys of 1-month-old mice. B and C, H&E staining of kidney sections from 1-month-old WT and CKO mice (B), and the number of glomeruli was counted (C). Scales bar represent 300 μm. D and E, *Ufl1* depletion in kidney of P0 CKO mice was validated by western blotting (D) and RT–qPCR analysis (E).

**Figure S2.** **YAP does not affect the expression of UFMylation components.** A, high-throughput sequencing data of kidney tissue from control group and *Yap*/*Taz* double-knockout embryonic mice. Data are extracted from GSE181400. B and C, after knockdown of *YAP* expression *via* siRNA in HK-2 cells, mRNA (B) and protein (C) levels of UFMylation components were detected by RT–qPCR and western blotting.

**Figure S3.** **Deletion of UFL1 enhances YAP phosphorylation in several kinds of cells.** A, B and C, Western blot analysis of phospho-YAP (Ser127), phospho-YAP (Ser397), phospho-LATS1/2 and phospho-MOB1 levels in 3T3-L1 cells (A), NCM460 cells (B) and HESCs (C) with depleted of UFL1 by siRNAs.

**Figure S4.** **UFL1 deletion in HK-2 cells by siRNAs or shRNAs.** A and B, UFL1 deletion in HK-2 cells by siRNAs (A) or shRNAs (B) was validated by western blotting.

**Figure S5.** **UFSP2 deletion increases YAP phosphorylation and degradation.** A, the effect of UFSP2 on YAP UFMylation *in vitro* was detected by western blotting. B, the phospho-YAP (Ser127), phospho-YAP (Ser397), phospho-LATS1/2 and phospho-MOB1 levels in HK-2 cells with loss of UFSP2 were detected by western blotting. C, the mRNA levels of YAP targets (*AREG*, *AXL*, *MYC*, *CTGF*, and *CYR61*) in UFSP2 deletion HK-2 cells were analyzed by RT–qPCR. D, Flag-YAP stability in HEK-293T cells with deletion of UFSP2 was examined by western blotting. The cells were treated with 100 μg/ml CHX for the indicated times. The graph represents quantification of the Flag-YAP protein levels (right panel).

**Figure S6.** **Flag-YAP-WT and Flag-YAP-K76R overexpression in HK-2 and HEK-293T cells.** A, Flag-YAP-WT and Flag-YAP-K76R overexpression in HK-2 cells was validated by western blotting. B, Flag-YAP-WT and Flag-YAP-K76R overexpression in si-Ctrl or si-UFSP1 HK-2 cells was validated by western blotting. C, Flag-YAP-WT and Flag-YAP-K76R overexpression in sg-Ctrl or sg-UFSP1 HEK-293T cells was validated by western blotting. **Table S1. List of reagents and resource.**

| **REAGENT or RESOURCE** | **SOURCE** | **IDENTIFIER** |
| --- | --- | --- |
| **Antibodies** | | |
| Phospho-YAP (Ser127) Rabbit mAb | Cell Signaling Technology | Cat# 13008 |
| Phospho-YAP1 (Ser397) Polyclonal antibody | proteintech | Cat# 29018-1-AP |
| YAP1 Rabbit pAb | ABclonal | Cat# A1002 |
| TEAD1 Rabbit pAb | ABclonal | Cat# A13366 |
| TEAD2 Polyclonal antibody | proteintech | Cat# 21159-1-AP |
| TEAD3 Polyclonal antibody | proteintech | Cat# 13120-1-AP |
| TEAD4 Polyclonal antibody | proteintech | Cat# 12418-1-AP |
| 14-3-3 (pan) Rabbit mAb | Cell Signaling Technology | Cat# 95422 |
| β-TrCP Rabbit mAb | Cell Signaling Technology | Cat# 4394 |
| Ubiquitin Antibody | Santa Cruz | Cat# sc-8017 |
| Anti-UBA5 antibody | abcam | Cat# ab177478 |
| UFC1 Polyclonal antibody | proteintech | Cat# 15783-1-AP |
| Anti-UFSP2 antibody | abcam | Cat# ab185965 |
| UFL1 Rabbit pAb | Oasis Biofarm | Cat# OB-PRB036-01 |
| UFSP1 Antibody | Santa Cruz | Cat# sc-398577 |
| UFM1 Rabbit mAb | ABclonal | Cat# A20875 |
| Anti-DDRGK1 antibody produced in rabbit | Sigma | Cat# HPA013373 |
| phospho-LATS1-T1079+LATS2-T1041 Rabbit mAb | ABclonal | Cat# AP1517 |
| LATS1 Polyclonal antibody | Proteintech | Cat# 17049-1-AP |
| LATS2-Specific Polyclonal antibody | Proteintech | Cat# 20276-1-AP |
| Phospho-MOBKL1B (Thr12) Polyclonal antibody | Proteintech | Cat# 29027-1-AP |
| MOB1A/B Rabbit mAb | ABclonal | Cat# A23963 |
| DYKDDDDK tag Recombinant monoclonal antibody | proteintech | Cat# 80801-2-RR |
| HA tag Polyclonal antibody | proteintech | Cat# 51064-2-AP |
| GAPDH Monoclonal antibody | proteintech | Cat# 60004-1-Ig |
| Rhodamine (TRITC)–conjugated Goat Anti-Rabbit IgG(H+L) | proteintech | Cat# SA00007-2 |
| HRP-Goat Anti-Mouse IgG | Abbkine | Cat# A25012 |
| HRP-Mouse Anti-Rabbit IgG | Abbkine | Cat# A25022 |
| **Bacterial and virus strains** | | |
| DH5α competent cell | Vazyme | Cat# C502-02 |
| BL21(DE3) competent cell | Vazyme | Cat# C504-02 |
| **Chemicals, peptides, and recombinant proteins** | | |
| DMEM Medium | Gibco | Cat# 11995065 |
| MEM Medium | MeilunBio | Cat# MA0217 |
| Fetal bovine serum (FBS) | VISTECH | Cat# SE100-B |
| Protease Inhibitor Cocktail (EDTA-Free) | Selleck | Cat# B14001 |
| Total RNA Isolation Reagent | Biosharp | Cat# BS259A |
| HiScript II Q RT SuperMix for qPCR | Vazyme | Cat# R222-01 |
| ChamQ Blue Universal SYBR qPCR Master Mix | Vazyme | Cat# Q312-02 |
| Lipofectamine 6000 Reagent | Beyotime | Cat# C0526 |
| Polyethylenimine (PEI) | Polysciences | Cat# 23966 |
| MG132 | Selleck | Cat# S2619 |
| Cycloheximide (CHX) | Selleck | Cat# S7418 |
| Anti-DYKDDDDK (Flag) Affinity Gel | Selleck | Cat# B23102 |
| Anti-HA magnetic beads | Selleck | Cat# B26202 |
| Glutathione Beads | Smart-Lifesciences | Cat# SA008025 |
| Crystal Violet Staining Solution | Beyotime | Cat# C0121 |
| CellTiter 96® Non-Radioactive Cell Proliferation Assay (MTT) | Promega | Cat# G4000 |
| Penicillin–streptomycin | Biosharp | Cat# BL505A |
| DAPI Staining Solution | Biosharp | Cat# BL105A |
| ATP | Cell Signaling Technology | Cat# 9804 |
| Phenylmethylsulfonyl fluoride (PMSF) | Beyotime | Cat# ST505 |
| BSA Albumin Fraction Ⅴ | BioFroxx | Cat# 4240GR100 |
| IPTG | BioFroxx | Cat# 1122GR025 |
| Recombinant Human His6-Ufc1 Protein | R&D Systems | Cat# E2-675 |
| Recombinant Human His6-UFM1 Activating Enzyme (UBA5) | R&D Systems | Cat# E-319 |
| Recombinant Human His6-UFM1 Protein | R&D Systems | Cat# UL-500 |
| **Critical commercial assays** | | |
| Hematoxylin and Eosin Staining Kit | Beyotime | Cat# C0105 |
| IHC detection kit | OriGene | Cat# PV-9001 |
| DAB Horseradish Peroxidase Color Development Kit | OriGene | Cat# ZLI-9019 |
| **Experimental models: cell lines** | | |
| HK-2 | National Science & Technology Infrastructure Center of China | Cat# 4201HUM-CCTCC00152 |
| HEK-293T | National Science & Technology Infrastructure Center of China | Cat# 3101HUMGNHu17 |
| **Recombinant DNA** | | |
| LV10-U6-puro-shNC | GenePharma | Cat# C06005 |
| LV10-U6-puro-shUFL1 | GenePharma | Cat# C06005 |
| pcDNA3.1-Vector | Invitrogen | Cat# V79020 |
| pcDNA3.1-DDRGK1 | This paper | N/A |
| pSG5-HA-Vector | Lab stored | N/A |
| pSG5-HA-UFM1 (WT) | This paper | N/A |
| pSG5-HA-UFM1 (∆C2) | This paper | N/A |
| pSG5-HA-UFM1 (∆C3) | This paper | N/A |
| pSG5-HA-UBA5 | This paper | N/A |
| pSG5-HA-UFC1 | This paper | N/A |
| pSG5-HA-UFL1 | This paper | N/A |
| pCDNA3.0-3×Flag-6×His-Vector | Lab stored | N/A |
| pCDNA3.0-3×Flag-6×His-YAP | This paper | N/A |
| pCDNA3.0-3×Flag-6×His-YAP (K76R) | This paper | N/A |
| pGEX-6P-1-Vector | GE Healthcare | Cat# 27-4597-01 |
| pGEX-6P-1-YAP | Zhao Bin | N/A |
| pGEX-6P-1-UFL1 | This paper | N/A |
| pGEX-6P-1-DDRGK1 | This paper | N/A |

**Table S2. List of siRNA sequences.**

| **Gene** | **Sense (5'-3')** | **Antisense (5'-3')** |
| --- | --- | --- |
| *YAP1-Homo* #1 | CAGGAAUUGAGAACAAUGATT | UCAUUGUUCUCAAUUCCUGTT |
| *YAP1-Homo* #2 | CCACCAAGCUAGAUAAAGATT | UCUUUAUCUAGCUUGGUGGTT |
| *UFSP1-Homo* #1 | CCAACUCCUUCUACAACCUTT | AGGUUGUAGAAGGAGUUGGTT |
| *UFSP1-Homo* #2 | GGAGAGGCUUUACUCGCACTT | GUGCGAGUAAAGCCUCUCCTT |
| *UFL1-Homo* #1 | GGAACUUGUUAAUAGCGGATT | UCCGCUAUUAACAAGUUCCTT |
| *UFL1-Homo* #2 | GAGGAGUAAUUUUUACGGATT | UCCGUAAAAAUUACUCCUCTT |
| *UFSP2-Homo* #1 | GCUGUCUUCAAACGCCCUUTT | AAGGGCGUUUGAAGACAGCTT |
| *UFSP2-Homo* #2 | GCUGAAGACCUGCAAGUUATT | UAACUUGCAGGUCUUCAGCTT |

**Table S3. List of sgRNA sequences.**

| **Gene** | **Sequence (5'-3')** |
| --- | --- |
| *UFL1-Homo* | CCAGCGGGCGCAGTTCGCCG |
| *UFSP1-Homo* | TACGTGGCAGAGGCGTCCCT |
| *UFSP2-Homo* | GTTGCTCCAGAAGAAACATG |

**Table S4. List of primers sequences used for RT-qPCR (Mus musculus).**

| **Gene**  **(Mus musculus)** | **Primer sequence (5′-3′)** |
| --- | --- |
| *Ufl1* | f: CTGGGACAACTGATTGATGAGAA |
|  | r: AGGAAGGTCATAGGCTTTACACA |
| *Yap1* | f: TCCGAAATCTTGGACGTGGA |
|  | r: GGCAGAGAAAAGCGGAACAA |
| *Areg* | f: AACTCTCCACAGGGGACTACG |
|  | r: CTTCTGTCTTGTTTTTCTTGGGC |
| *Axl* | f: ATGGCCGACATTGCCAGTG |
|  | r: CGGTAGTAATCCCCGTTGTAGA |
| *Myc* | f: CCTTTGGGCGTTGGAAACC |
|  | r: CGTCGCAGATGAAATAGGG |
| *Ctgf* | f: AGTGTGCACTGCCAAAGATG |
|  | r: CCAGGCAAGTGCATTGGTAT |
| *Cyr61* | f: GCTCAGTCAGAAGGCAGACC |
|  | r: GTTCTTGGGGACACAGAGGA |
| *Tafa5* | f: AACCTGTGAGATTGTGACCCT |
|  | r: TCGAGTGGTGCCTGCTATC |
| *Cited1* | f: ATGCCAACCAGGAGATGAAC |
|  | r: TGGCAGTAGGAGAGCCTGTT |
| *Pla_2_g_7_* | f: ACAACTCCTGCAAGCTGGAAT |
|  | r: AAGTAAGTTGCCGATGCAGA |
| *Pax8* | f: GCCATGGCTGTGTAAGCAAG |
|  | r: GTTCTGCCGCTTGTAGTCTCC |
| *Lim1* | f: GCACGTCAAGTGCGTCCAG |
|  | r: GAGAGCTGCTTGTTACACATCATG |
| *Six2* | f: CAAGAATGAAAGCGTGCTCA |
|  | r: CTTCTCCGCCTCGATGTAGT |
| *Sall1* | f: GTGAGCGGCTGATGTTTG |
|  | r: CTTGGTTGACCCTTCTCTGT |
| *Pax2* | f: CCCACATTAGAGGAGGTGGA |
|  | r: GATGTGCTCTGATGCTTGGA |
| *β-actin* | f: GACATGGAGAAGATCTGGCA |
|  | r: GGTCTCAAACATGATCTGGGT |

**Table S5. List of primers sequences used for RT-qPCR (Homo sapiens).**

| **Gene**  **(Homo sapiens)** | **Primer sequence (5′-3′)** |
| --- | --- |
| *UFL1* | f: GCATGTTCAGTTAGTGTTGGGA |
|  | r: TGATATGGTGACCTGACCACTTT |
| *YAP1* | f: CCTGCGTAGCCAGTTACCAA |
|  | r: CCATCTCATCCACACTGTTC |
| *AREG* | f: TCTAGTAGTGAACCGTCCTCG |
|  | r: TTTGGGGGGGCTTAACTACC |
| *AXL* | f: AACCTTCAACTCCTGCCTTCTCG |
|  | r: CAGCTTCTCCTTCAGCTCTTCAC |
| *MYC* | f: CCCGCTTCTCTGAAAGGCTCTC |
|  | r: CTCTGCTGCTGCTGCTGCTGGTAG |
| *CTGF* | f: GCTTACCGACTGGAAGACACG |
|  | r: CGGATGCACTTTTTGCCCTT |
| *CYR61* | f: CTTACGCTGGATGTTTGAGTGT |
|  | r: AGACTGGATCATCATGACGTTCT |
| *UFSP1* | f: GGAGAGGCTTTACTCGCACTTC |
|  | r: CCAATACCAGGACATAGGCTTCC |
| *UFSP2* | f: GAAAGCGGAGGACACCATTATG |
|  | r: ACCAGGAGTTTACGAACTTTTCC |
| *UFM1* | f: CAGTGTTCCTGAAAGTACACCTT |
|  | r: CCGCAGTTCTGAACCATGTTTTA |
| *UBA5* | f: ATGGCATTGAAACGAATGGGA |
|  | r: CAACACCTACTATTGCTACGGC |
| *UFC1* | f: TGACAACGATTGGTTCCGACT |
|  | r: GGGGCAGTAGTAGGATATGTGAT |
| *DDRGK1* | f: GGTTGTGCTCTTGGAAGACCT |
|  | r: TCACACCTGTTATAGTCCCCTC |
| *β-actin* | f: CAAAGTTCACAATGTGGCCGAGGA |
|  | r: GGGACTTCCTGTAACAACGCATCT |
